# A Review of the Impact of EFL Teachers’ Affectivity and Surgency and Learners’ Shyness on Their Language Attainment

**DOI:** 10.3389/fpsyg.2022.916432

**Published:** 2022-05-23

**Authors:** Tian Tian

**Affiliations:** School of Foreign Languages, East China Normal University, Shanghai, China

**Keywords:** extroversion, surgency, EFL teachers, affectivity, shyness

## Abstract

Teaching is considered as a complex and demanding profession affected by a number of student-related and teacher-related factors. Instructors’ personality trait has been long scrutinized as an influential issue, which can facilitate or impede the learning process. Teachers’ affective factors and personality types (e.g., surgency) are among the most commonly studied aspects of educational research, particularly English as a Foreign Language (EFL) teachers. The review of related studies suggests that extrovert teachers are more likely to encourage EFL learners to pursue their educational objectives and master the target language effectively because they can create friendly and positive learning conditions where learners are engaged in classroom activities. This will be primarily prominent for EFL learners with higher levels of shyness. Such students prefer to remain reticent during the course, evade engaging in collaborative tasks, and tend to use a number of avoidance strategies while facing a stressful situation. Hence, language teachers are recommended to employ a variety of techniques as well as appropriate tasks in order to help these pupils overcome their negative affective characteristics so that they can enjoy learning the content and producing the target language in a facilitating and inspirational atmosphere.

## Introduction

Given the communicative and collaborative nature of the teaching/learning process, teachers’ and learners’ personality traits, emotions, and other affective variables should be regarded as crucial determinants of a successful teaching/learning experience (Brown, 2007; Tejeda et al., 2016). With the advent of humanistic psychology, affective factors started to gain as much attention as the cognitive factors had already received (Wang, 2005; Lei, 2007). In fact, the significance of personality variables and the consensus on learners as dynamic and affective human beings led to a great number of studies and theories highlighting the need to evaluate and implement such factors while developing teaching materials and selecting the teaching methods to be applied in the classroom. Similarly, it was argued that second/foreign language learning process can also be highly influenced by personality and affective factors, particularly because of the communicative nature of language learning. English, as an international language has been taught all over the world; hence, it is crucial for English as a Foreign Language (EFL) teachers to be familiar with these psychological dimensions of language learning so that EFL learners can achieve the optimum amount of the materials and internalize their understanding of future implementation (Mohammadian, 2013; Kırkaǧaç and Öz, 2017; Noreen et al., 2019; Ahmadi-Azad et al., 2020). The introduction of the Big Five personality model by Eysenck and Eysenck (1985) is considered a critical attempt to include teachers’ and learners’ personality variables into the learning process. According to MacIntyre et al. (2019), one of the primary aspects of the model was the introversion/extraversion (also known as surgency) dimension, which should be perceived as if individuals might be gifted with a degree of each side. In particular, extraversion reflects people’s desire to integrate into the community. For instance, John et al. (2008) believed that extroversion is associated with being involved in social settings, promoting positive emotions, and building confidence (Malki, 2020). Hence, highly extroverted people are likely to be understood as energetic, verbose, and friendly individuals (Deniz and Satici, 2017).

It is also noteworthy that the affective filter hypothesis (Krashen, 1985) assumes that learners will be able to comprehend the input optimally provided that they are experiencing facilitating emotional situations. For instance, learners need to have high motivation, low stress, and anxiety, as well as appropriate self-confidence (Baptiste, 2005). Furthermore, Schwartz (2005) and Thornbury (2005) contended that negative psychological factors, like fear of mistake, shyness, and anxiety may have adverse impacts on learning and particularly the demonstration of the learned content. Shyness refers to the restraining and anxious state of individuals in the presence of others. It is regarded as a feeling of anxiety and restraint in places where others are (Jones et al., 1986). Henderson and Zimbardo (1993) defined shyness as the experience of distress and reticence, which hinders the achievement of interpersonal goals. It is associated with avoidance tendencies and obsession with oneself while facing social conventions. Similarly, researchers argued that learners’ potential might not be sufficient for the transition from their present status to the desired level (i+1, according to the premises of Krashen’s input hypothesis); thus, teachers’ supportive role, i.e., the scaffolding function, should be implemented appropriately in order to facilitate learners’ progress (Vacca, 2008; Zheng, 2016). Moreover, Chang (2015) believed that affectivity is concerned with any attempt to save the other interlocutor’s face and avoid causing difficulties or resentment. It is also applicable in teaching, where teachers are required to establish a positive and supportive relationship with learners in order to create a friendly atmosphere and help them improve and become autonomous.

Regarding the educational settings, instructors and teachers are expected to demonstrate positive affective emotions so that learners would attempt to overcome their negative emotions, including anxiety and shyness. In a seminal study, Moafian and Ghanizadeh (2009) contended that teachers’ major task might be to convey knowledge to the learners; nonetheless, they can play even more prominent roles in the learning process through developing an optimal environment, improving learners’ motivation and self-confidence, as well as implementing appropriate teaching methods and approaches tailored at student’s needs and learning styles (Williams and Burden, 2000; Anderson, 2004). Moreover, researchers claimed there is a strong relationship between extroversion and affective factors in education and psychology. Consequently, surgency and immediacy are among the most influential affective factors teachers can implement to provide a friendly and supportive learning atmosphere for the learners (Zuckerman, 2005; Swenddal, 2011; Shabani and Ghasemian, 2017).

Eventually, teachers’ affectivity is also regarded as a constructive and empowering element throughout their careers. For instance, Meyer and Allen (1984) underlined the prominence of affective commitment and defined it as the affirmative feeling of engagement and immersion into one’s job. Consequently, such committed employees are more likely to implement their sense of belonging so as to identify and deal with potential barriers at work and seek organizational objectives (Carmeli, 2003). In addition, it is argued that highly extroverted individuals are more likely to demonstrate greater performance at work and commitment to the organization. Tallman and Bruning (2008) asserted that these employees will pursue the psychological contract, which guarantees their efforts and engagement in their duties while working with their fellow colleagues.

Nonetheless among the bulk of research on teachers’ and learners’ personality characteristics and emotions, the teaching/learning literature has witnessed only some studies, particularly observing the relationship between teachers’ emotional factors and learners’ shyness (Rebecca Chu, 2008; Alavinia and Salmasi, 2012; Chen et al., 2015; Ahsan et al., 2020). Therefore and respecting the prominence of the influence of individual differences on EFL learning outcome, the present study attempts to review the related articles to identify the most appropriate approach to the impact of instructors’ surgency and affectivity on shyness among learners.

## Theoretical Background

### Affectivity

According to Villamil and De Guerrero (1996), affectivity is associated with avoiding to cause any kind of negative feelings in others and proposing empathy instead, which might be expressed in the form of positive and supportive comments.

With regards to the implementation of affectivity in educational settings, Anderson (2004) highlighted instructors’ capabilities to moderate and improve learners’ academic experiences, leading to the achievement of their educational objectives. Therefore, teachers are capable of developing and enhancing students’ self-esteem through the provision of scaffolding behaviors and a proper learning environment (Williams and Burden, 2000). Tschannen-Moran et al. (1998) proposed that teachers’ affectivity refers to their perception of the skills and aptitudes to manage the learning environment and successfully present the course content to particular pupils. In the meantime, students’ character, including efficacy and inspiration may also influence teachers’ effectiveness and their belief in their potential.

Furthermore, Moafian and Ghanizadeh (2009) contended that the nature and extent of the teacher-student relationship should be of paramount importance in the academic setting. They also argued that language learning courses, particularly English as a foreign language, are mostly planned to include a number of creative and pair/group tasks, where instructors’ responsiveness and support play crucial roles accordingly. Since instructors intend to identify and involve students’ emotions (by promoting positive feelings such as self-confidence and autonomy and addressing negative feelings like anxiety and shyness) in the learning process, this is called reflective teaching. In a similar vein, Lei (2007) claimed that English language teachers are expected to manage the classroom and provide adequate linguistic material to the students provided that it is uncommon for Chinese students to engage in authentic conversations with native speakers of English and to be familiar with their culture. In addition, Alibakhshi (2011) examined the role of teachers’ personalities and gender in their preferences over teaching approaches and techniques. This study summarized that extroverted-introverted, judging, and sense were among the most common personality characteristics of EFL teachers in Iran.

Eventually, the findings of Lei’s (2007) seminal study revealed that the following affective factors have influenced students in the EFL classroom: teachers’ personality, expertise, classroom management, morality, assessment procedures, rapport, and teaching approaches. Therefore, teachers should be impartial and supportive to each student because they can enhance their motivation to make greater efforts and achieve their academic goals. As a result, it is recommended that teachers refrain from making behavioral and attitude distinctions between great and poor students. In fact, they should intend to support and encourage the weak students by highlighting their potential to help them feel important and succeed. Therefore, these are the characteristics of a good teacher.

### Extroversion (Surgency)

According to Hosford and Martin (1980), the term “surgency” was first proposed by Philip Hosford, reflecting individuals’ distinctive features that inspire other people’s evaluation of their capabilities and behaviors. Barrick et al. (1993) then explained that the term “extraversion” can be used interchangeably with “positive emotion” and “surgency.”

Extraversion was demonstrated as a passionate tendency to the social environment associated with positive emotions (e.g., enthusiasm and happiness), decisiveness (i.e., being able to lead a social group), and involvement (i.e., offering energy and excitement while communicating with others) (John et al., 2008). In a similar vein, McCrae and Costa (2008) proposed that extraversion entails six aspects, including warmth, openness, encouraging emotions, assertiveness, engagement, and a zest for excitement. Besides, extroverted people are regarded as having great self-esteem, which is likely because of the implementation of appropriate problem-solving strategies while facing challenging events in life (Mathews et al., 2009).

According to Malki (2020), extroversion represents one’s engagement with the outside world and the perception of eagerness. Furthermore, Malouff et al. (2005) claimed that extroversion includes a range of emotions and manifestations from shyness and isolation to eager contribution and enjoyment-seeking behaviors. Hence, it was asserted that the extent and the magnitude of social relationships are crucial to the concept of extroversion. In contrast, introversion reflects individuals’ attitudes toward independence and reticence (Baptiste, 2018).

On the other hand, Helgoe (2008) argued that introversion reflects individual’s attitudes toward seclusion rather than solidarity. Introverted people would prefer to rely on themselves to deal with challenges in life, and they are more likely to avoid sharing their problems in groups except to a close friend. Consequently, they will be anxious if faced with a dynamic issue associated with making a spontaneous decision, which might result in isolation and the demonstration of uncanny behaviors.

Similarly in the field of education, it is recommended to revisit the role of teacher’s personality in teaching/learning experiences. For instance, Shabani and Ghasemian (2017) contended that extroverted teachers are highly enthusiastic and outgoing so that they can build friendly and supportive relationships with learners. They will also employ a variety of techniques and approaches in order to encourage and engage students in the classroom.

According to Dost et al. (2017), English language teachers’ personality has been proved more influential compared to their cognition. They concluded that extroverted teachers will be able to manage their classrooms more successfully. Besides, Thomason (2011) argued that such instructors attempt to build effective relationships with their fellow students, which is highlighted in their sociable tendency. EFL teachers, the extroverted in particular, are enthusiastic, active, and innovative; hence, they prefer to implement learner-centeredness in their classes and tend to improve language learning students’ self-efficacy and autonomy through requiring a variety of teamwork and pair work tasks in the classroom. Finally, extroverted teachers aim to create an enjoyable and energetic environment for language learners to motivate linguistic and communicative engagement in the classroom (Jalili and Mall-Amiri, 2015).

Briggs-Myers and Briggs (1985) as well as Eysenck and Eysenck (1964) developed distinctive and popular scales to measure individuals’ personality characteristics, particularly extroversion/introversion dichotomy. Myers-Briggs Personality Type Indicator (MBTI) is regarded as one of the widely used personality tests, which has been employed by a great number of researchers (Ehrman and Oxford, 1988; Altunel, 2015; Mahdavi Zafarghandi et al., 2016; Dost et al., 2017). The A form was outlined in 1943; then, it was revised and the second edition of the MBTI Manual was published in 1985. The M form of this instrument (1998), which has been in vogue recently, entails 93 items on Extroversion/Introversion, Sensing/Intuition, Thinking/Feeling, and Judging/Perceiving personality traits. Moreover, the internal consistency of the instrument was reported at 0.88 for the Middle East, which indicated an acceptable reliability index (Mahdavi Zafarghandi et al., 2016). Furthermore, Eysenck Personality Inventory (EPI) has been already implemented in a variety of contexts (Chen et al., 2015; Jalili and Mall-Amiri, 2015; Mozaffari and Ghodratinia, 2015; Shabani and Ghasemian, 2017). The EPI questionnaire encompasses 57 items, and the respondents are required to answer either yes or no. This scale evaluates extroversion (24 items), neuroticism (24 items), and sociability (9 items) (Dibah and Marashi, 2013).

### Shyness

As pioneering practitioners in psychological issues, Brodt and Zimbardo (1981) proposed that some people are mentally affected by others’ opinions and assessments of their personality and behaviors. Therefore, these people, who are referred to as shy individuals, constantly avoid having contact with others, where they may come across harsh and negative comments. Shyness is also regarded as a restricting factor against building relationships and initiating interactions (Rebecca Chu, 2008; Tang and Schmidt, 2017). Zimbardo further introduced different types of shyness, including introverted and extroverted shy people. The underlying distinction between these two types of shyness resides in the fact that, unlike introverted shy people, extroverted ones do not necessarily try to avoid taking part in group activities. Instead, they often enjoy their interactions with others; however, extroverted shy people mainly argue that they feel disregarded.

In addition, Cheek and Bauss (1981) also defined shyness as an instance of social anxiety that threatens people’s self-awareness and –confidence in public. According to Wadman et al. (2008), shyness is considered as a persistent characteristic, which is associated with strain, inhibition, and uneasiness while contacting others. Consequently, shyness refers to the tendency to escape the potential feeling of discomfort and worriedness in social conventions (Tong et al., 2011).

Moreover, Doyon (2000) contended that shyness can easily affect even clever students in the classroom. They may already know the correct response to the teachers’ question, yet they intend to remain reticent because of the fear of being mocked or ridiculed by their peers. Taylor (2001) believed that Rogers was one of the pioneering scholars to put humanistic psychology, and particularly empathy and respective positive notions, into practice in 1960s. Since the concept of the humanistic approach has been introduced to the field of education, the primary objectives of pedagogical planning and teaching methodology have been based on the notion of learners as individual human beings with distinctive cognitive and affective characteristics. As a result, learner-centeredness was associated with the new trend in educational contexts, where learners’ emotions and thoughts were the main features of student development. Lei (2007) further argued that humanistic teachers attempt to reduce the impact of learners’ negative emotions such as shyness and anxiety as well as develop their positive emotions like efficacy and enjoyment.

Arnold and Brown (1999) argued that affective factors such as shyness and anxiety are likely to hinder language learners’ attainment. Such EFL learners may opt to remain silent in the classroom because of the fear of making mistakes and being evaluated as inattentive and stupid. According to Liu (2006) second/foreign language learners may continue to feel shy or anxious throughout the course; besides, their negative feelings would be highlighted when faced with a demanding and interpersonal task in the classroom. Hence, language learners may be easily affected by the English language teachers’ or peers’ reactions in the classroom, which might lead to making mistakes frequently or remaining reticent as an avoidance strategy (Middleton and Frank, 2009).

Furthermore, shyness is believed to affect the language learning process negatively, particularly when language learners are required to produce the language and communicate with their teachers or classmates. In a similar vein, Baldwin and Caroline (2011) asserted that engaging in verbal interactions is more likely to develop anxiety and shyness among language learners. Therefore, it will be essential to promote learners’ communicative competence and encourage them to implement their English knowledge despite the probable mistakes and difficulties (Ahsan et al., 2020).

## Empirical Studies

### Teachers’ Affectivity and Surgency

Teachers’ characteristics, including personality and affective factors, have proved influential on learners’ motivation and achievement, particularly in EFL/ESL classes. Moreover, it is crucial for teachers to promote social interactions with students to develop their learning capacity. For instance, Eryilmaz (2014) argued that a well-adjusted emotional and affective personality can help teachers develop a lively and inspirational learning environment. Meanwhile, the study confirms the effectiveness of teachers’ extroversion and friendliness in students’ success, especially adult learners.

MacIntyre et al. (2019) aimed to assess the correlation between language teachers’ personality and well-being and stressful situations and stressors. Forty-seven EFL/ESL university instructors participated in this study. They employed the big five personality scale (including extroversion), the PERMA wellbeing framework, and a stressor scale to measure these variables. The results of the study indicated a positive relationship between teachers’ personality traits and their well-being; nonetheless, no association was found between personality traits and stressors. Furthermore, Garcia et al. (2011) employed the big five inventory to examine the correlation between teachers’ personality type and secondary students’ attainment. They concluded that teachers’ affective factors can have a substantial impact on learners’ achievement.

Jalili and Mall-Amiri (2015) used Eysenck Personality Inventory to evaluate female teachers’ extroversion/introversion characteristics. Then, they selected thirty extroverted and thirty introverted instructors teaching English in private institutes in Tehran, Iran. This study aimed to assess EFL teachers’ class management based on their personality type. The findings demonstrated a significant difference between the two groups of teachers, where extroverted teachers reported performing more proficiently in managing EFL classes. Similarly, Dost et al. (2017) confirmed that extroverted teachers were more successful in class management; besides, they concluded that students in the extroverted teacher group showed an outstanding perception of the course content and were highly motivated to learn English compared to the students in the introverted teacher classroom.

It is also noteworthy that Binti (2014) asserted that psychologists believe attitudes, inspiration, perceptions, and capabilities form individuals’ personalities. Therefore, that study was conducted on 37 graduate teaching students to examine the effectiveness of personality types on academic achievement. According to Eysenck Junior Personality Inventory, respondents’ personality characteristics were significantly correlated with their academic success; in particular, the impact of extroversion was approved in this regard.

Mozaffari and Ghodratinia (2015) investigated the impact of teachers’ personality type (extroversion/introversion) on elementary students’ English language attainment. For this purpose, they allocated 25 students to two distinct classes: a fifteen-session semester with an introverted teacher and another fifteen-session semester that was taught by an extroverted teacher. According to the students’ performance in their final exam, the researchers concluded that the mean score of students who were taught by an extroverted teacher was greater than those taught by an introverted teacher. Eventually, they argued that teachers’ friendly and sociable characteristics have encouraged elementary learners to involve attentively and improve their scores. In addition, Khalid et al. (2015) conducted a study on 210 elementary school teachers in Pakistan with the aim of identifying the impact of instructors’ background and behavior on learners’ understanding of coursebook contents. They concluded that these teachers were able to create collaborative classroom environments, where they could address students’ mistakes in a supportive and friendly manner. Nonetheless, it was found that teachers’ behavior did not have a substantial effect on students’ learning.

In a similar vein, Shabani and Ghasemian (2017) aimed to evaluate the role of teachers’ personalities in the type and frequency of techniques employed while teaching pronunciation. For this purpose, they decided to use Eysenck Personality Inventory to assign the sixty participants into two groups of extroversion and introversion. Then, they were asked to complete another questionnaire regarding the implementation of various techniques to teach pronunciation in EFL classes. The findings indicated that there is a significant distinction between instructors’ extroversion and introversion and their choice of teaching approaches. Besides, it was reported that extroverted teachers implemented a wider range of pronunciation techniques.

### Learners’ Shyness

Shyness is believed to affect learners’ understanding and performance in the classroom, it is particularly influential among foreign language learners. In this regard, Tong et al. (2011) conducted research investigating the effectiveness of shyness on elementary students’ Chinese and English vocabulary skills. They studied 54 EFL learners in terms of their perception of expressive and receptive vocabulary. The findings revealed that there is a relationship between shyness and students’ understanding of English as a foreign language and Chinese as their mother tongue. Pazouki and Rastegar (2009) intended to explore the role of extroversion/introversion and shyness in students’ proficiency. They selected 93 EFL university students in Kerman, Iran, and asked them to complete both the Eysenck Personality Questionnaire and Stanford Shyness Inventory. They found that shyness and EFL proficiency (such as vocabulary and reading comprehension) are not correlated. Besides, they concluded there is an adverse relationship between extroversion and shyness.

Shyness has been reviewed in relation to different language skills. For instance, Ostovar Namaghi et al. (2015) performed a seminal study to investigate the impact of shyness on EFL learners’ speaking proficiency. For this purpose, a sample of 165 middle school students in Semnan province, Iran was considered as the study population. They were asked to respond to the Persian version of Zimbardo’s revised scale to evaluate the extent of students’ shyness. The research findings indicated that shyness is negatively correlated with students’ speaking performance. They further concluded that the relationship was more significant among female learners. Similarly, Ahsan et al. (2020) observed the role of shyness in EFL learners’ speaking skill among 200 college and university students in South Punjab. They employed a structured researcher-made questionnaire to determine the degree of shyness. The results of the study revealed that there is an association between shyness as well as lack of confidence and learning spoken language as well as EFL students’ speaking performance. Although the relationship was negative, it was not significant. Eventually, Babapoor et al. (2018) examined the potential relationship between shyness and EFL learners’ oral proficiency. Fifty female learners, studying English at a private institute in Urmia, Iran, took part in this study. Then, the Revised Cheek and Buss Shyness Scale (RCBS) was implemented to measure the degree of shyness among the participants. Besides, the respondents were asked to describe a picture through a narrative oral task. The results showed that EFL learners’ shyness is significantly and negatively associated with the accuracy and fluency of their speeches.

Furthermore, the influence of shyness on EFL learners’ motivation and willingness to communicate was the major concern of Mohammadian’s (2013) research. She studied sixty learners taking English courses in a private institute in Shiraz, Iran, and the Revised Cheek and Buss Shyness Scale (RCBS) was used to assess the degree of shyness among these students. The findings indicated that shyness may mainly affect learners’ intrinsic motivation. Moreover, it was reported that shyness does not have a significant impact on these learners’ willingness to communicate. Rebecca Chu (2008) examined the association between shyness and EFL learning, with regards to learners’ willingness to communicate, motivation, and anxiety. For this purpose, a sample of 364 university students taking English courses in Taiwan was selected by the researcher. Then, the 13-item Revised Cheek and Buss Shyness Scale (Cheek, 1983) as well as a self-rated shyness questionnaire were employed to assess the learners’ level of shyness. In terms of the relationship between shyness and foreign language anxiety, the findings suggest a relatively positive correlation. Besides, it was found that shyness and willingness to communicate are negatively correlated among these Chinese students since shy students tend to remain reticent and refrain from engaging in conversations due to the fear of possible mistakes or judgment by others. Similarly, Chien-Tzu (2006) aimed to observe the potential correspondence between EFL learning and shyness among college students in Taiwan. The results revealed that despite the negative relationship between shyness and foreign language learning, students’ shyness did not significantly hinder their language attainment (e.g., listening, reading, and speaking).

Oflaz (2019) conducted a study on 110 students, learning German as a foreign language. As a part of the study, the researcher aimed to investigate the impact of anxiety and shyness on these learners’ speaking proficiency and academic success. For this purpose, he employed a revised version of Cheek’ shyness scale that contains 20 items and is scored on a Likert-scale. The findings suggested that anxiety was significantly correlated with academic achievement; however, there was a moderate relationship between students’ shyness and their academic success, which is in line with the findings of another study by Vinitwatanakhun (2019). Oflaz (2019) further contended that learners’ shyness increases as they perceive higher levels of anxiety in the classroom. Finally, he claimed that anxiety and shyness would predict nearly 25% of foreign language students’ academic achievement.

Butt et al. (2011) conducted a correlational study on 150 middle-school students in Pakistan and examined the relationship between shyness and self-esteem. They employed a 25-item shyness questionnaire developed by Vanaja et al. (2004). According to the findings of this study, there was a positive but insignificant association between learners’ shyness and their self-esteem. Meanwhile, they asserted that the combination of low self-esteem and shyness is likely to develop introversion among students, which might lead to a sense of resentment and concern regarding their potential academic failure. What is more, Alavinia and Salmasi (2012) performed a seminal study to examine the relationship between Iranian EFL learners’ shyness and language learning attitudes. They used a 44-item standard questionnaire to assess the level of shyness among 104 EFL learners in Urmia, Iran. It is also noteworthy that language learning attitudes were evaluated based on five different subcategories, including self-image, inhibition, risk-taking, ego-permeability, and ambiguity tolerance. The results of the study revealed that none of these language learning attitudes were significantly affected by learners’ shyness. In the end, they concluded that language learners’ gender might have a significant impact on their manifestation of shyness as well as learning attitudes.

Eventually, in an attempt to scrutinize the relationship between shyness and learning adjustment (the tendency to create a balance in learning settings), Chen et al. (2018) conducted a comprehensive study on 670 high-school students in China. They used the 31-item Shyness Scale for Chinese Middle School students to measure the degree of shyness among these students. The results of the study highlight the significant negative relationship between shyness and learning adjustment.

## Implications and Suggestions

Personality characteristics can determine the extent of individuals’ diverse behaviors and thoughts in similar occasions (Chamorro-Permuzic and Furnham, 2010). These personality differences can lead to a variety of personal, occupational, and social outcomes accordingly. It has been approved that teachers’ personality can play a crucial role in students’ success because it affects different dimensions of teaching and learning practices. For instance, Rockoff (2004) asserted that learners’ academic attainment can be facilitated by their teachers’ personal and professional qualities. Moreover, Polk (2006) claimed that teachers can improve their occupational competence and effectiveness through understanding and empowering their personality traits, such as extroversion, conscientiousness, surgency, openness, etc. As a result, teachers will be able to choose and embark on the most appropriate teaching methods tailored to their personal preferences and personality type, which should be further reflected in their students’ academic achievement (Dost et al., 2017).

Similarly, some seminal studies attempted to evaluate the predictive power of teachers’ personality traits on their own professional achievement as well as their students’ academic achievements. For instance, Hakimi et al. (2011) concluded that teachers’ personality characteristics can contribute to nearly 50% of their learners’ academic attainment. Besides, Kırkaǧaç and Öz (2017) employed Big Five personality model to investigate the influence of EFL teachers’ characteristics on their professional achievement. They further argued that EFL instructors’ personality traits can predict 17% of their academic success in the teaching profession. As a result, it is critical to identify and revisit teachers’ personality dispositions so that universities and colleges can design appropriate and efficient curricula accordingly. Raising instructors’ awareness (including EFL teachers) of their instincts and characters should lead to highly informed decisions in the classroom and the development of effective teacher-learner interactions with respect to their personality traits.

Thomason (2011) claimed that classroom management can be affected by various factors, including teachers’ personality. Extroversion/Introversion dichotomy has been regarded a critical personality trait among teachers and students, and there are a great number of studies investigating the nature, impact, and promotion of such characteristics. He further concluded that due to the desire to build social interactions with their colleague as well as the students, extrovert teachers might have the required capabilities to manage the classes more efficiently. Accordingly, Dost et al. (2017) highlighted the extrovert teachers’ classroom management skills, particularly English teachers, because they are more likely to implement learner-centered teaching approaches where the learners are motivated to engage in classroom activities. The development of students’ autonomy will lead to a positive classroom atmosphere and encourages students to follow their academic objectives and respect classroom rules more effectively. It is also noted that extrovert teachers should be able to create collaborative and lively conditions to help their students master the course content accordingly. In addition, teachers with higher degrees of extroversion will provide their learners with the opportunity to comment on the quality and type of the course content; on the contrary, introverted teachers are more likely to take charge of the teaching material and teaching approaches (Bayazidi and Behnam, 2013).

Furthermore, Shabani and Ghasemian (2017) asserted that extrovert teachers’ energetic and interactive nature will lead to the implementation of a wider range of teaching techniques and respective academic tasks, which prepare the ground for the learners to indicate their perception of the course content in a way that is more appropriate according to their personal preferences and learning styles. Consequently, it is crucial for managers and teacher trainers to highlight these personality traits and attempt to promote a variety of materials and approaches based on such individual differences between teachers. Consistent with the findings of related studies, the researchers believe that instructors’ personality characteristics can play influential roles to help learners, particularly EFL learners, get along with the teaching materials and classroom environment. As a result, it is imperative for language teachers to identify their personal capabilities and their pupils’ preferences in order to employ the teaching methods tailored at their students’ needs as well as cognitive and emotional characteristics. Moreover, English teachers should perceive the necessity to implement communicative approaches and create a lively and friendly atmosphere in the classroom so as to enhance the students’ linguistic intake.

On the other hand, learners’ affective factors can also play a critical role in their academic success. Positive (e.g., enjoyment, self-confidence, motivation) and negative (e.g., anxiety, shyness, and boredom) affective characteristics are both equally prominent in leading to constant attainment. Ostovar Namaghi et al. (2015) claimed that despite their significance, some affective factors have not been well received by the educational systems, managers, and even teachers. For instance, learners’ shyness might be disregarded because of these learners’ tendency to remain silent as an avoidance strategy to being scolded or judged negatively (Oxford, 1999; Allvar, 2003). Hence, there is a need for further analysis of such personal characteristics across various cultures and learning settings.

Rebecca Chu (2008) argued that culture may also play a crucial role in the manifestation of personal differences even in similar situations. For example, because of particular moral issues such as self-discipline, Chinese people mostly tend to keep silent at social events, which is also observable in educational settings, including EFL classes. Nevertheless, the communicative and collaborative nature of language courses may require language learners to be highly engaged in a variety of classroom tasks, such as pair work or other group activities. Consequently, instructors, particularly language teachers, should have enough expertise and capabilities to provide lively and friendly learning conditions to foster optimal involvement among all the students. Vinitwatanakhun (2019), who investigated the correlation between shyness and EFL students’ language achievement, argued that it is unwise to disregard the critical impact of culture on students’ performance. Although the findings revealed a moderate relationship between shyness and academic success among Thai students, the researchers highlighted the influence of culture by proposing to EFL teachers in Thailand to consider the reticent and shy nature of the Thai population as an embedded personality trait. Consequently, EFL instructors are recommended to implement various teaching approaches (e.g., pair work tasks), tolerate the Thai students’ delayed responses, and ignore learners’ minor communication mistakes in order to motivate such students to keep producing the target language, particularly before their classmates.

In a similar vein, Ahsan et al. (2020) asserted that EFL teachers need to prepare the classroom tasks so that learners, especially the shy students, feel confident to take part actively and attentively and to have enough knowledge and then adequate time to figure out the optimum solution for educational tasks. EFL learners should be able to produce the target language knowing that the teacher’s or peers’ reactions are not detrimental, even if they may make frequent mistakes.

In addition, Mohammadian (2013) argued that EFL teachers can implement a variety of learning tasks in the classroom to encourage shy students to get engaged in the learning process. According to the findings of the study, she noted that shy EFL learners have mainly opted to participate in group discussions rather than interpersonal meetings or lectures. That is because group discussions in language classrooms in Iran are held in a way that not all the participants are required to be equally involved in the discussion. Hence, even shy students can enjoy participating in a group task, which may help improve their self-confidence and motivate them to be much more active in future occasions. Similarly, Butt et al. (2011) concluded that motivation and praise can help learners deal with shyness and low self-confidence. Consequently, the researcher reckons that language teachers should be knowledgeable enough to identify shy students and then to employ appropriate techniques to facilitate their immersion into the classroom activities that will lead to academic attainment. Riasati and Rahimi (2018) studied the influential factors on EFL learners’ willingness to communicate. The findings indicated a moderate correlation between shyness and willingness to communicate; nonetheless, they argued that students’ silence in the classroom cannot be only attributed to their level of shyness. Language learning classrooms are required to provide a welcoming and encouraging setting for the students in order to obtain the maximum learner contribution. Hence, even shy students would perceive the classroom environment as friendly and innovative so they can voice their ideas and communicate with their peers without the fear of judgment and negative reactions.

In the end, it is recommended to perform cross-cultural studies to examine the role and effectiveness of teachers’ personality types as well as learners’ affective factors. As a result, researchers might be able to propose innovative and influential approaches and ideas so that teachers can express their capabilities more appropriately and learners will be inspired to take charge of their learning process and achieve their academic objectives.

## Data Availability Statement

The original contributions presented in the study are included in the article/supplementary material, further inquiries can be directed to the corresponding author/s.

## Ethics Statement

The studies involving human participants were reviewed and approved by the East China Normal University Academic Ethics Committee. The patients/participants provided their written informed consent to participate in this study.

## Author Contributions

The author confirms being the sole contributor of this work and has approved it for publication.

## Conflict of Interest

The author declares that the research was conducted in the absence of any commercial or financial relationships that could be construed as a potential conflict of interest.

## Publisher’s Note

All claims expressed in this article are solely those of the authors and do not necessarily represent those of their affiliated organizations, or those of the publisher, the editors and the reviewers. Any product that may be evaluated in this article, or claim that may be made by its manufacturer, is not guaranteed or endorsed by the publisher.
